# From La Gazette Médical

**Published:** 1869-01-15

**Authors:** 


### FOREIGN ITEMS.

[FROM LA GAZETTE MÉDICAL.]

M. Claude Bernard has recently performed some interesting experiments in illustration of pathogenesis. He injected into the veins of rabbits, amygdaline or emulsine separately; the animals appeared to suffer no inconvenience. After which he injected into the different veins of the same animals amygdaline and emulsine, and the animals speedily manifested the symptoms of hydrocyanic acid poisoning, with fatal termination. The amygdalic fermentation is thence produced in the blood, since the emulsine a fermentescible matter, is brought into contact with its ferment.

In another series of experiments, M. Bernard injected into the veins of a dog, a solution of sugar and of yeast of beer. The animal died in two or three days with adynamic symptoms; the autopsy revealed lesions analogous to those of putrid infection. Here, again, the primitive alteration of the blood has had no other than the result of the fermentation of the sugar under the influence of the yeast. It would have been interesting to have ascertained, by microscopic examination, if the presence of the torula cerevisiæ would confirm the induction already made from direct observations.

---

M. A. Dubreuil communicates to the *Journal de l'Anatomie et de la Physiology, &c., &c., Ch. Robin*, the following note upon the cicatrization of bones and nerves.

Having removed from rabbits the middle portion of a radius with its periosteum and having seen the bone reproduced, he compared these results with those obtained after the resection of nerves, and deducts therefrom the following conclusions, viz: "When a portion of the bone or of nerve is resected, the loss of substance is replaced by a tissue which, at the end of some time, undergoes *osseous or nervous substitution*, for which it is not at all necessary that the normal sheath of the portion excised, whether periosteum or neurilemma, should remain," etc., etc., etc.

---

M. J. O. A. Mongeot asserts, as the result of his investigations into certain derangements of nutrition consecutive to nervous affections, that there exist no fibro-cellular trophic nervous elements or tubes having direct action upon the nutrition and the secretion of the elements situated in the vicinity of their extremities or of their cells of origin.

There are no trophic nerves known other than the vaso-motors.

The secretory disturbances, those of absorption, indurations, softenings and hypertrophics or other alterations consecutive to lesions of nerves, are consequences of circulatory perturbations by the intermediation of the aforesaid nerves, and not the consequence of the action of the nerves which would have, after the manner of electricity for example, an influence upon the molecular or chemical acts of assimilation or disassimilation in a zone of a certain extent outside of their surface.

---

Tubercles of the retina and the choroid, considered as diagnostic of tuberculous meningitis.

Apropos of this subject M. Bouchut asserts :

1st. That tubercles of the retina and of the choroid indicate either tuberculous meningitis or general tuberculosis.

2d. A febrile case presents disturbances of intellect, of motion and of sensation, the existence of tuberculous meningitis may be inferred.

3d. Tubercles of the choroid constitute one of the most rare manifestations of the tuberculous diathesis.

4th. Tubercles of the choroid occur under the form of white miliary granulations. Sometimes brilliant and nacreous.

5th. The retrogression granulo-fatty metamorphosis of the normal elements of the retina and of the choroid cells, is the origin of tubercles of the retina and of the choroid.

